# Engineering an Oxygen‐Binding Protein for Photocatalytic CO_2_ Reductions in Water

**DOI:** 10.1002/anie.202215719

**Published:** 2023-04-04

**Authors:** Yunling Deng, Sudharsan Dwaraknath, Wenhao O. Ouyang, Cory J. Matsumoto, Stephanie Ouchida, Yi Lu

**Affiliations:** ^1^ Department of Chemistry University of Texas at Austin Austin TX 78712 USA; ^2^ Department of Chemistry University of Illinois at Urbana-Champaign Urbana IL 61801 USA

**Keywords:** Artificial Metalloenzyme, CO_2_ Reduction, Cobalt, H_2_ Evolution, Photocatalysis

## Abstract

While native CO_2_‐reducing enzymes display remarkable catalytic efficiency and product selectivity, few artificial biocatalysts have been engineered to allow understanding how the native enzymes work. To address this issue, we report cobalt porphyrin substituted myoglobin (CoMb) as a homogeneous catalyst for photo‐driven CO_2_ to CO conversion in water. The activity and product selectivity were optimized by varying pH and concentrations of the enzyme and the photosensitizer. Up to 2000 TON(CO) was attained at low enzyme concentrations with low product selectivity (15 %), while a product selectivity of 74 % was reached by increasing the enzyme loading but with a compromised TON(CO). The efficiency of CO generation and overall TON(CO) were further improved by introducing positively charged residues (Lys or Arg) near the active stie of CoMb, which demonstrates the value of tuning the enzyme secondary coordination sphere to enhance the CO_2_‐reducing performance of a protein‐based photocatalytic system.

## Graphical abstract

We report a homogeneous photocatalytic system based on cobalt porphyrin substituted myoglobin (CoMb) for CO_2_ to CO conversion in water. By optimizing the reaction conditions, our catalysts achieved up to 2000 TON(CO) at low enzyme concentrations, and a product selectivity of ∼ 80 % with an increased enzyme loading. We show that the efficiencies of CO generation and overall TON(CO) can be improved by introducing positively charged residues near the active site of CoMb.

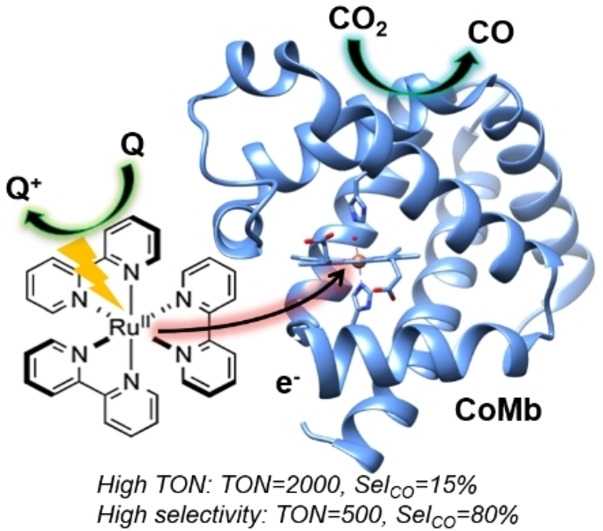

## Introduction

Conversion of CO_2_ into feedstock chemicals is a major endeavor in the global strategy to achieve net zero carbon emission.^[1]^ Toward this goal, decades of research have generated many homogeneous and heterogeneous catalysts for CO_2_ reduction. While many catalysts that are capable of high turnover frequencies (TOF) have been produced, most of them do so at the cost of low product selectivity of CO against the competing reduction of proton to H_2_. In contrast, enzymes such as nickel carbon monoxide dehydrogenase (Ni‐CODH) can convert CO_2_ to CO with not only high TOF of 12 s^−1^, but also 100 % selectivity for CO.^[2]^ It has also been recently discovered that a reductase component of nitrogenase from *Methanosarcina acetivorans*, called the Fe protein, can also reduce CO_2_ and convert it hydrocarbons.[^3^, ^4^] However, structural features characteristic to the native enzymes that are responsible for CO_2_ reduction are not fully understood. To address this issue, we and others have taken a bottom‐up approach of engineering artificial enzymes to mimic the active‐site structures, and thus activities of native enzymes,[^5^, ^6^, ^7^, ^8^, ^9^, ^10^, ^11^, ^12^, ^13^] complementing the top‐down approach of studying native enzymes. In addition to validating findings from native enzymes and their variants, the bottom‐up engineering approach can reveal structural features that may not be obvious when studying native enzymes, which may be applicable to other catalyst designs. Moreover, engineering artificial enzymes based on small and stable protein scaffolds compared with utilizing complex native enzymes for CO_2_ reduction would benefit the scale up in future applications, especially if they can be expressed in vivo.^[4]^

Metalloenzymes for CO_2_ reduction achieve such impressive catalytic metrics in large part by using precise, asymmetric, and non‐covalent interactions in the secondary coordination sphere (SCS) to activate CO_2_ and lower the energy of the transition states along the target reaction pathway.[^14^, ^15^] To understand the roles of these structural features, mutations of residues in the SCS of native proteins, such as nitrogenase MoFe proteins, where amino acid substitutions at the key residues (α‐70^Val^ and α‐195^His^) near the FeMo‐cofactor, have been shown to significantly alter the product distribution of CO_2_ reduction by controlling CO_2_ and proton access.[^16^, ^17^] In addition to modifying native enzymes, artificial metalloenzymes, where abiotic catalysts, such as Co‐porphyrin,^[18]^ [Ni^II^(cyclam)]^2+^,^[19]^ and Ni‐terpyridine,^[20]^ have been incorporated in protein scaffolds for CO_2_ reduction. For example, double tyrosine mutations near the Ni‐terpyridine binding site significantly enhanced the CO_2_ reduction rate, presumably by facilitating proton transfer.^[20]^ Despite the progress made, the structure‐function relationship is still not well understood. To accelerate the design‐build‐test‐learn cycle for an engineered catalyst, we chose myoglobin (Mb), as it has already been characterized and modified extensively in the literature. Mb is an O_2_‐binding protein that is small, stable, and highly soluble, which makes it substantially easier to crystallize and amendable for modifications with natural and unnatural amino acids, in addition to replacing the native heme with other metallocofactors.[^21^, ^22^, ^23^, ^24^] While engineering Mb for various other chemical transformations have been reported,[^6^, ^7^, ^8^, ^9^, ^25^, ^26^, ^27^, ^28^, ^29^, ^30^, ^31^, ^32^] Mb has not been applied to CO_2_ reduction. On the other hand, metalloporphyrins have been discovered as both photocatalysts and electrocatalysts for CO_2_ reduction.[^33^, ^34^, ^35^, ^36^, ^37^, ^38^, ^39^, ^40^, ^41^, ^42^, ^43^, ^44^, ^45^, ^46^, ^47^, ^48^, ^49^] Many of these molecular porphyrin‐based catalysts have been modified with pendant groups to gain insights into the SCS on CO_2_ reduction activities.[^42^, ^44^, ^45^, ^50^, ^51^, ^52^, ^53^] Since the SCS is known to contain precise, asymmetric, and non‐covalent interactions, it has been extremely challenging to synthesize metalloporphyrin complexes that are rigid enough to precisely position multiple weak non‐covalent interactions asymmetrically around the metalloporphyrin, and with high enough yield for studies. In comparison, heme proteins such as Mb can be readily prepared in a few days with higher yield than chemically synthesized metalloporphyrins. More importantly, the rigid 3D structure of proteins allows precise placement of multiple asymmetric non‐covalent interactions around the heme center, with minimal variation of either synthesis yield or structures. On the other hand, photocatalytic CO_2_ reduction using cytochrome (cyt) *b*_562_ to support a Cobalt(II) protoporphyrin IX (CoPPIX) cofactor has been reported,^[18]^ but both the activity and product selectivity were moderate, probably because the work explored only the roles of primary coordination sphere in conferring and tuning the activity and selectivity, and did not take the full advantages of using protein scaffolds to modulate SCS. To address these issues, we herein report an engineered myoglobin in which the native heme is replaced with CoPPIX and demonstrate that this CoMb is capable of photocatalytically catalyzing the reduction of CO_2_ to CO in the presence of [Ru(bpy)_3_]^2+^, with up to 2000 TON and up to ∼ 80 % product selectivity, the highest among engineered enzymes, after optimizations by varying pH, concentrations of the enzyme and photosensitizer, as well as introducing positively charged residues (Lys or Arg) in the second sphere.

## Results and Discussion

Since a low oxidation state of the metal center is typically required for the reduction, we sought to take advantage of the [Ru(bpy)_3_]^2+^ photosensitizer that has been widely used to promote the photocatalytic CO_2_ to CO conversion in aqueous solution by other catalysts.[^18^, ^19^, ^33^, ^54^, ^55^] The photoexcitation of [Ru(bpy)_3_]^2+^ to [Ru(bpy)_3_]*^2+^ can be quenched by a reductant (ascorbate), resulting in a highly reducing Ru^I^ with a reported reduction potential (*E*°′) of [Ru(bpy)_3_]^2+/1+^ being −1.26 V vs. SHE.[^54^, ^55^] We mixed Mb with an excess amount of [Ru(bpy)_3_]^2+^ photosensitizer and sodium ascorbate, a sacrificial reductant, and surveyed the headspace for CO and other C_1_ and C_2_ short‐chain hydrocarbons through GC‐FID. This experiment showed no evidence of CO_2_ conversion.

**Figure 1:**
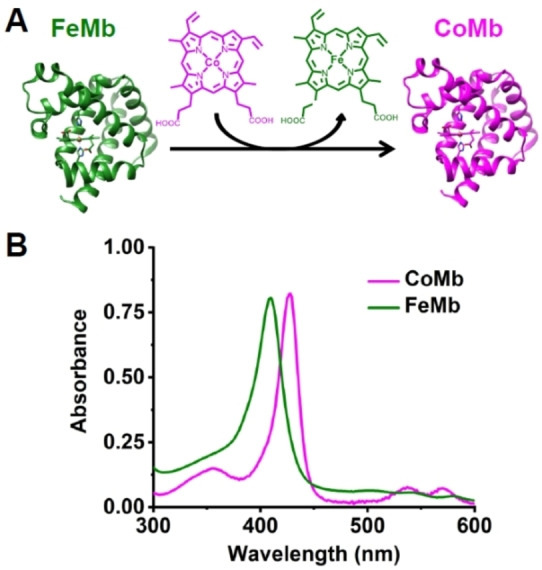
A) Scheme showing the substitution of the native heme *b* in Mb with CoPPIX. B) Absorption spectra of Fe(III)Mb and Co(III)Mb measured at pH 7.

As an alternative to the heme (FePPIX), CoPPIX is known to display reduction potentials that are hundreds of millivolts higher than the corresponding values for FePPIX (e.g., *E*°′(Co^I/II^PPIX)≅−1.1 V and *E*°′(Fe^I/II^PPIX)≅−1.3 V vs. SHE),^[56]^ offering a higher chance to access low valent Co species capable of reducing CO_2_ than FePPIX. Therefore, we replaced the native heme *b* in Mb with CoPPIX using a previously reported protocol.^[57]^ As shown in Figure 1, the Soret band in the UV/Vis spectrum shifted from 409 nm in native FeMb to 427 nm in CoMb, with a similar shift in the Q‐bands around 550 nm. These shifts in the UV/Vis spectra are consistent with the heme in Mb being replaced with CoPPIX. Mass spectrometry of CoMb, shown in Figure S11A, not only confirmed the mass of the protein scaffold but verified the incorporation of a cofactor with the same mass as CoPPIX. Furthermore, a previously published high‐resolution crystal structure of Co‐substituted sperm whale Mb confirmed that the CoMb closely resembled the native Mb,^[58]^ indicating minimal structural perturbation from the metalloporphyrin substitution.

With CoMb prepared, we proceeded to test whether CoMb could reduce CO_2_ photocatalytically. Using [Ru(bpy)_3_]^2+^ as a photosensitizer and sodium ascorbate as a sacrificial reductant, the headspace samples from the reaction vials after photoirradiation were analyzed by gas chromatography (GC). As shown in Figure S1, a new peak around 3.2 min. retention time was observed upon the photoreduction and mass spectrometry of this peak indicated it being CO. In contrast, no such a peak was observed when the experiment was repeated in the absence of [Ru(bpy)_3_]^2+^, ascorbate, or CoMb (Figure S2), suggesting that the photosensitized reduction of CoMb is required for generation of the CO. Moreover, no CO was observed when the CoMb enzyme was replaced with the free CoPPIX metallocofactor, indicating that the protein scaffold is required for a functional catalyst. To confirm that CO_2_ is the origin of CO production, we performed the experiment using ^13^C‐labeled CO_2_ and measured the GC‐MS spectrum of the headspace gas. Two peaks with *m*/*z*=29 eluted around the retention time for CO (Figure S3). One of the two peaks was present in the absence of enzyme, and even in a sample of air, and so it is likely ^29^N_2_ that is present naturally in air. The other peak, which is observed only when all reaction components are present, must be ^29^CO, confirming that CoMb is capable of reducing CO_2_ to CO.

To quantify the reaction rates and turnovers, GC calibration curves of CO and H_2_ were obtained (see Figure S4). Then, we measured the amount of CO generated in the headspace and found it to gradually increase over the course of the two‐hour photoirradiation, ultimately amounting to >1000 turnovers in the presence of 0.1 μM WT CoMb (Figure S5A). H_2_ evolution reaction (HER) is known as a competing reaction for CO_2_ reduction in solution.^[59]^ Moreover, CoMb has been reported to photocatalytically reduce protons into H_2_ gas in presence of [Ru(bpy)_3_]^2+^.^[60]^ To find out the product selectivity of our enzyme, we used GC‐TCD to quantify the H_2_ that is evolved along with CO produced. As shown in Figure S5B, an even higher amount of H_2_ was produced by CoMb than CO.

**Figure 2:**
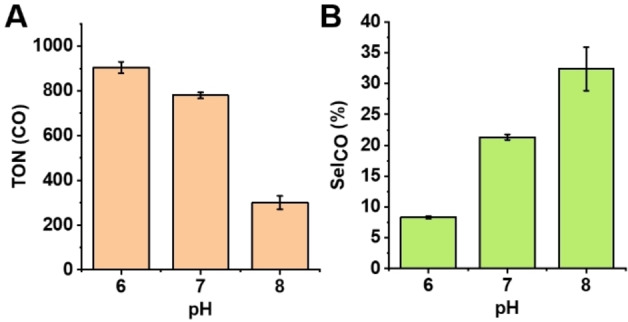
pH dependence of CO production and product selectivity. A) TON of CO under irradiation with blue LED light for an hour in 100 mM ascorbic acid, 1 mM [Ru(bpy)_3_]^2+^, and 1 M potassium phosphate buffers at pH 6, pH 7, and pH 8. B) Product selectivity (Sel_CO_) under the same conditions. Experiments were performed in triplicate; the error bars are calculated from the standard deviations of the data.

To determine the optimal reaction conditions for CO_2_ reduction and to suppress the HER, we first examined the pH effects on both CO_2_ and proton reduction activities by performing the reactions under three different pH conditions, pH 6, 7, and 8 (Figure 2). By increasing the pH from 6 to 7, the proton reduction activity was significantly suppressed without compromising the CO_2_ reduction activity. The product selectivity (Sel_CO_) is about two‐fold higher at pH 7 compared to that at pH 6. The higher product selectivity towards CO generation under elevated pH resulted from the lower availability of protons in solution for HER. When the pH was further increased to 8, both CO_2_ and proton reduction activities dropped, although product selectivity increased. The lower activity at pH 8 was initially attributed to the lower CO_2_ concentration in solution since the conversion of bicarbonate into CO_2_ becomes unfavorable at high pH. However, when CO_2_ gas was used as the source of CO_2_ instead of bicarbonate, comparably low TON for CO and H_2_ was observed (Figure S6), suggesting that the low activities at pH 8 is due to the intrinsic properties of those reductions, where protons are required for both CO_2_ reduction and HER. Since we observed both high TON and product selectivity at pH 7, we performed all following assays at pH 7 to investigate the roles of other factors in tuning the activity and product selectivity.

**Figure 3:**
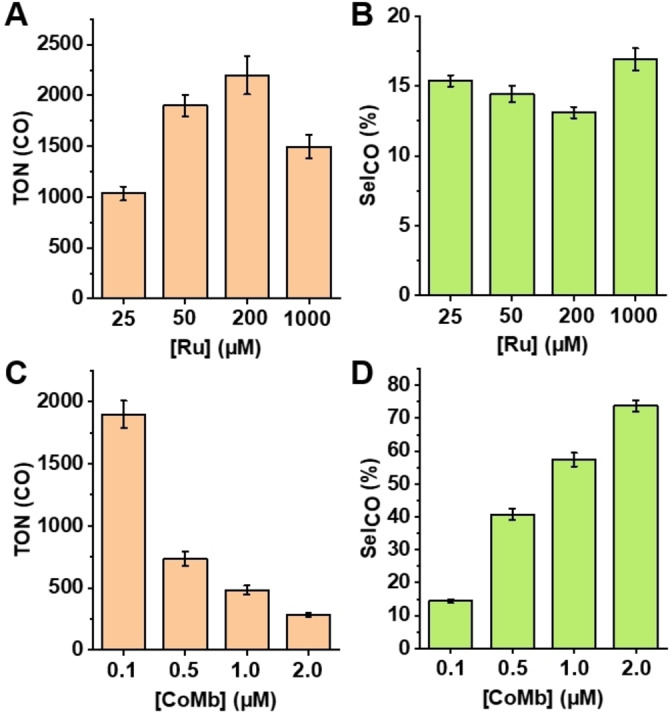
TON of CO and Sel_CO_ upon 2 hours of photoirradiation with WT CoMb at varying concentrations of [Ru(bpy)_3_]^2+^ and CoMb at pH 7. A), B) The concentration of [Ru(bpy)_3_]^2+^ was varied from 25 μM to 1 mM, while the enzyme concentration was 0.1 μM. C), D) The concentration of enzyme was varied from 0.1 μM to 2 μM, while the [Ru(bpy)_3_]^2+^ concentration was 50 μM.

Following our studies on the effects of pH, we investigated the influence of enzyme and photosensitizer concentrations to determine the optimal conditions for CO_2_ reduction. The production of CO and H_2_ was followed over the first two‐hour photoirradiation (Figure S7). We first varied the concentrations of the photosensitizer [Ru(bpy)_3_]^2+^ from 25 μM to 1 mM, while holding the enzyme concentration constant at 0.1 μM. As shown in Figure 3A, increasing the concentration of [Ru(bpy)_3_]^2+^ up to 200 μM enhanced TON(CO). These results are expected, since the reduction of substrate relies on the electron transfer from the photogenerated reducing species of the photosensitizer, and lower [Ru(bpy)_3_]^2+^ concentrations limit the overall rate of photocatalysis. When the concentration of [Ru(bpy)_3_]^2+^ was further increased to 1 mM, the TONs of both the CO and H_2_ decreased, which is likely due to the saturation of the active reducing agent, Ru^I^, given the limited number of photons that passed through the sample. Overall, the product selectivity under different concentrations of [Ru(bpy)_3_]^2+^ was in the range of 15–30 %, and the selectivity decreased with increasing time of photoirradiation, reaching 15 % at around 2 hours (Figures 3B and S7C). Since the TONs for CO at 50 μM and 200 μM [Ru(bpy)_3_]^2+^ are similar, we chose 50 μM [Ru(bpy)_3_]^2+^ for the subsequent investigations.

To determine the optimal enzyme concentration, the concentration of CoMb was varied from 0.1 μM to 2 μM, while keeping all other conditions constant (Figures 3C and D). Interestingly, 0.1 μM enzyme at 2 h photoirradiation reached 2000 TON(CO), which is more than two folds higher than the TON(CO) produced by 0.5 μM enzyme (Figure 3C), suggesting that lower enzyme concentration produced higher TON. This observation holds true for both CO and H_2_ (Figures S7D and E). More importantly, as the enzyme concentration increased from 0.1 to 2 μM, the selectivity for CO (Sel_CO_) improved (Figure 3D), reaching ∼ 80 %, which has rarely been achieved for photocatalytic CO_2_ reduction in water.[^33^, ^61^, ^62^] It is interesting that the TON(CO) and CO product selectivity are inversely correlated, and that this correlation depends on catalyst concentration. As shown in Figures 3C and D, when the CoMb catalyst concentration is increased, the selectivity for CO product is also increased, but the TON(CO) decreased. When the catalyst concentration is increased with photosensitizer concentration held constant, the ratio between the catalyst and the photosensitizer becomes higher; thus the number of reducing equivalents that each molecule of catalyst can receive during the same period of time is reduced due to the limited number of photons and the quantum yield of the photosensitizer. As a result, the TON(CO) of CoMb is decreased at higher catalyst concentrations. Moreover, the efficiency of electron transfer from the reducing Ru^I^ species to the catalyst is increased at higher catalyst concentrations, resulting in less reactive Ru^I^ species remaining in solution to promote H_2_ evolution reactions and thus the product selectivity toward CO is increased. A similar result was observed in a previous study using modified cobalt porphyrin molecular catalysts.^[33]^ Under an extreme condition where no enzyme was provided, the production of H_2_ was drastically higher and CO generation was considerably lower than those observed in the presence of 2 μM CoMb (Figure S8). This result strongly suggests that the HER is primarily catalyzed by the photosensitizer itself, rather than CoMb. Photocatalytic H_2_ evolution by ruthenium bipyridyl complexes has literature precedence, with generation of H_2_ presumably catalyzed by degradation of [Ru(bpy)_3_]^2+^.[^63^, ^64^] To search further evidence for the photosensitizer degradation, the electronic absorption spectra of the reaction before and after the photoirradiation were compared. As shown in Figure S9, the absorbance in the visible region, which is dominated by the metal‐to‐ligand charge transfer band of [Ru(bpy)_3_]^2+^ decreased after photoirradiation, which strongly indicates photosensitizer degradation. These results are consistent with previous studies on the photochemical stability of [Ru(bpy)_3_]^2+^, where the photodegradation of [Ru(bpy)_3_]^2+^ led to the loss of one bipyridyl ligand and its substitution with an anionic ligand from the solution.[^65^, ^66^, ^67^]

To examine whether the observed degradation of the photosensitizer could be a limiting factor for the overall TON(CO) of our photocatalytic system, we added the same amount of fresh photosensitizer after the first 2 h photoirradiation. However, we did not observe any recovery of photocatalytic activity. As protein photodegradation is not an uncommon issue,^[68]^ we replenished the system with both fresh enzyme and photosensitizer at the end of the 2 h photoirradiation and only observed 10 % recovery of the activity. These results indicate that there are still other factors limiting the performance of our photocatalytic system. One possible factor is product inhibition by CO, which has been reported to occur in native enzymes.^[69]^ To test this hypothesis, we performed activity assays with a mixture of CO_2_ and CO gas and obtained around 40 % loss of the total TON(CO) upon 2 h photoirradiation, suggesting that product inhibition is a limiting factor in photocatalysis.

To characterize liquid‐phase products, we collected ^1^H NMR spectra on the reaction solution. The solution was lyophilized and redissolved in D_2_O to minimize the signals from water. The production of formate was quantified by comparing the integrated area of formate peak to that of the NMR internal standard, sodium 4,4‐dimethyl‐1‐silapentane‐1‐sulfonate (DSS). Upon 2 hours of photoirradiation, about 2.0–3.5 μmol formate was produced and its yield was higher in the absence of the enzyme (Figure S10). These results indicate that the formate production was mainly catalyzed by the degraded form of [Ru(bpy)_3_]^2+^ instead of the enzyme. Indeed, the carbonyl‐bound variant of the degraded [Ru(bpy)_3_]^2+^ have been proposed to catalyze CO_2_ to formate conversion.[^64^, ^70^]

**Figure 4:**
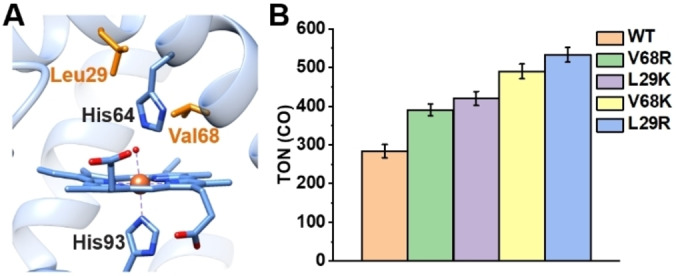
A) The crystal structure of WT Mb (PDB ID: 5YCE)^[73]^ with the active site residues that are mutated to Lys or Arg shown in orange color. B) TON of CO by WT CoMb and variants upon photoirradiation for 2 hours in the presence of 2 μM enzyme and 50 μM [Ru(bpy)_3_]^2+^ at pH 7.

It has been shown that Lys563 around the Ni‐CODH metal‐binding site play an important role in catalysis by stabilizing the partial negative charge on the O atoms of CO_2_ during activation.[^2^, ^71^] The incorporation of pendant amine groups has also been found to enhance CO_2_‐reducing activity in some synthetic catalysts.[^51^, ^72^] To determine whether such a secondary sphere feature can tune the CO_2_ reduction activity in our CoMb system, we individually replaced Leu29 and Val68, two conserved residues near the active site of Mb, with either Lys or Arg (Figure 4A). It was confirmed by CD spectroscopy that the protein secondary structure was not perturbed by either Co‐incorporation or point mutations (Figure S11B).

We first examined the mutational effects under high‐TON conditions with 0.1 μM enzyme and 50 μM [Ru(bpy)_3_]^2+^ but observed only minor variations in activity and product selectivity between mutants (Figure S12 and Table S1). In contrast, under high Sel_CO_ conditions all mutants exhibited higher TON(CO) than WT CoMb, with L29R and V68K mutants displaying an approximate two‐fold increase in TON(CO) after 2 hours of photoirradiation (Figure 4B). Along with increased CO generation, the HER was also accelerated by mutations, leading to comparable product selectivities among WT CoMb and mutants (Figures S13 and Table S2). One explanation for the enhanced TON(H_2_) in CoMb mutants is that the positively charged residues also stabilize cobalt hydride intermediates for H_2_ production. On the other hand, since HER can be catalyzed by the photosensitizer itself (Figure S8), it is difficult to differentiate the TON(H_2_) originated from the protein or the photosensitizer. Therefore, both TON(H_2_) and Sel_CO_ should be considered as evaluations of the photocatalytic system as a whole. In contrast, CO_2_RR is mainly catalyzed by CoMb enzymes, thus changes in TON(CO) are more valid reflections of the mutational effects on the catalytic performance of the enzymes. We monitored CO generation every 20 min over the course of 2 hours of photoirradiation (Figure S13), all mutants exhibited a higher TON(CO) compared to WT CoMb (Figure S13A). In particular, the fast increase in TON(CO) with L29R and V68K CoMb suggests that the catalytic rates of our catalysts were enhanced by these mutations. However, due to the limitations of photocatalytic systems that we discussed earlier, the increase in TON slowed down dramatically after 1 hour of photoirradiation for all the mutants and WT CoMb. We attribute the different mutational effects among the variants to the different positions and orientations of the positively charged side chain in stabilizing the partial negative charge on the oxygen atom of bound CO_2_ substrate. To corroborate our hypothesis that the enhanced CO_2_ reduction rate can be ascribed to the stabilizing effects exerted via positively charged residues, we prepared a V68D CoMb variant to introduce a negative charge near the active site. As a result, we observed slightly lower TON(CO) relative to WT CoMb (Tables S1 and S2). Interestingly, beneficial effects of the Arg/Lys mutations are observable only under high selectivity conditions. With a higher enzyme concentration under the high selectivity conditions, the total number of reducing equivalents each enzyme can receive in a given period of time is lower than that under high TON conditions due to limitations in the number of absorbed photons and the low quantum yield of the photosensitizer. Under such conditions, the stabilizing actions exerted by Arg/Lys residues become more crucial in prolonging the lifetime of the bound CO_2_ substrate (or COOH* intermediate) at the active site to achieve the two‐electron reduction to CO.

To evaluate the performance of our CO_2_ reducing enzyme, we compared both TONs(CO) and product selectivities of CoMb with other reported enzyme‐based photocatalytic systems (Table 1).[^18^, ^19^, ^20^] In comparison to the Co‐cyt *b*_562_ catalyst, where a similar enzyme construction strategy was employed (protein scaffold containing CoPPIX), our L29R CoMb achieved over 50‐fold higher TON(CO) and similar product selectivity under the high‐TON condition, despite a 4‐fold reduction in photoirradiation time. In fact, the TON(CO) of CoMb is even higher than most Fe‐ or Co‐based porphyrin catalysts performing in nonaqueous solutions.^[35]^ Under high‐selectivity conditions, our L29R CoMb catalyst generates a ∼ 15‐fold higher TON(CO) and near 4‐fold higher product selectivity than Co‐cyt *b*_562_. An azurin‐based (S78C‐RuCuAz‐[1]) and photosensitizer protein‐based (PSP2‐Ni(terpy)_2_) CO_2_‐reducing catalysts confer the highest product selectivity of 100 %. However, both catalysts display lower TONs(CO) of 4.6 and 75, respectively. It is interesting that both S78C‐RuCuAz‐[1] and PSP2‐Ni(terpy)_2_ systems employ a covalent linkage between the photosensitizer and catalyst, which results in 1 : 1 ratio during photocatalysis. According to the results of enzyme concentration dependent assays, it is likely that the higher selectivity of these systems is due to the increased efficiency in electron transfer from the active photosensitizer to the catalyst, which helps to minimize side reactions. However, the overall activity is compromised under such a high selectivity condition. Increasing the relative concentration of the catalyst with respect to the photosensitizer will reduce the productivity in a same period, since the only source of electrons for the catalyst is from the photosensitizer that it is covalently linked to. To overcome the current limitations in enhancing both activity and selectivity, more efforts need to be made towards optimizing the coordination environment (both primary and secondary) for more efficient CO_2_ reduction.

**Table 1:** TON for photocatalytic CO production by artificial CO_2_‐reducing metalloenzymes.

Mutant	pH	TON (CO)	Sel_CO_ [%]	Ref.
WT CoMb^[a]^	7	1900±100	14.4±0.6	this work
WT CoMb^[b]^	7	280±20	73.9±1.7	this work
L29R CoMb^[a]^	7	2000±100	13.3±0.3	this work
L29R CoMb^[b]^	7	530±20	77.7±0.3	this work
Co‐cyt *b*_562_	7	34±6^[c]^	20.6	[18]
S78C‐RuCuAz‐[1]	7.25	4.6±0.2	100	[19]
PSP2‐Ni(terpy)_2_	8	75^[d]^	100	[20]

[a] The reaction condition was 2 h photoirradiation at pH 7 in the presence of 0.1 μM enzyme and 50 μM [Ru(bpy)_3_]^2+^. [b] The reaction condition was 2 h photoirradiation at pH 7 in the presence of 2.0 μM enzyme and 50 μM [Ru(bpy)_3_]^2+^. [c] 8 hours irradiation. [d] 12 hours irradiation.

## Conclusion

In summary, we have developed a CoMb‐based biocatalyst for CO_2_ reduction that has attained >2000 turnovers for CO production, the highest among engineered enzymes. After optimizing the enzyme concentration, a more selective (Sel_CO_≈80 %) catalyst was also obtained. The TON of our catalysts was further improved by introducing Arg or Lys in the secondary coordination sphere, achieving two‐fold enhancement in CO production under high‐selectivity conditions. The design‐build‐test‐learn cycle for artificial enzymes can be greatly accelerated by tools such as directed evolution, genetic code expansion, and computational methods. We will utilize these tools to build on the first‐generation catalysts reported here and design the next generation of better CO_2_‐reducing catalysts in our CoMb scaffold.

## Conflict of interest

The authors declare no conflict of interest.

1

## Supporting information

As a service to our authors and readers, this journal provides supporting information supplied by the authors. Such materials are peer reviewed and may be re‐organized for online delivery, but are not copy‐edited or typeset. Technical support issues arising from supporting information (other than missing files) should be addressed to the authors.

Supporting Information

## Data Availability

The data that support the findings of this study are available in the Supporting Information of this article.
