# Supplementary material for: Engineering an Oxygen‐Binding Protein for Photocatalytic CO2 Reductions in Water
Source: Angew Chem Int Ed Engl. 2023 Apr 4;62(20):e202215719. doi: 10.1002/anie.202215719 (PMC10946749; doi:10.1002/anie.202215719)
Supplement: Supplementary file 1 — Supporting Information [file ANIE-62-0-s001.pdf]

Angewandte Chemie
Eine Zeitschrift der Gesellschaft Deutscher Chemiker

Supporting Information

Engineering an Oxygen-Binding Protein for Photocatalytic CO_2 Reductions in Water

*Y. Deng, S. Dwaraknath, W. O. Ouyang, C. J. Matsumoto, S. Ouchida, Y. Lu**

Materials and Methods

Unless otherwise noted, all chemicals and reagents were obtained from commercial suppliers (Millipore Sigma, VWR, Fischer Scientific) and were used without further purification.

Protein expression, purification, and heme substitution

All Mb variants was expressed and purified as reported.[1] Native heme *b* was replaced with cobalt protoporphyrin IX (FrontierSci) according to the Teale's butanone method with modifications.[2] Specifically, the protein was diluted in 100 mM sodium acetate pH 3.5 buffer and then the free heme was extracted with methyl ethyl ketone in a separatory funnel until the top organic layer was colorless. The bottom aqueous layer was collected and CoPPIX dissolved in DMSO was subsequently added. The solution was dialyzed against 100 mM potassium phosphate pH 7.0 buffer to remove the organic solvent. The reaction product was concentrated and passed down a PD-10 column to remove excess cobalt porphyrin. The mass spectrum of the cobalt-substituted protein was acquired on an Agilent 6546 Q-TOF mass spectrometer.

Determination of extinction coefficients of cobalt myoglobin

The molar absorptivity of CoMb variant was determined using a combination of optical absorption spectroscopy and ICP-MS, as reported previously.[3,4] The absorbance at 426 nm band of the CoMb was measured by UV-Vis using an Agilent 8453 diode-array spectrophotometer. The concentration of cobalt content in samples was determined by PerkinElmer NexION 350D ICP-MS cobalt element analysis. The extinction coefficient at 426 nm was then calculated based on those values.

Circular Dichroism Measurements

The circular dichroism (CD) spectra of WT Mb and CoMb mutants were acquired on a JASCO-J815 CD spectropolarimeter. To measure the far-UV (200−250 nm) CD spectra, proteins with a final concentration of around 3 μM were prepared in a 50 mM potassium phosphate buffer at pH 7. The pathlength of the quartz cuvette was 1 mm.

Photoreduction reaction setup

The reactions contain 0.1 μM protein, 1 mM $[Ru(bpy)_3]^{2+}$, 100 mM sodium ascorbate, and 100 mM sodium bicarbonate as CO_2 source in 1 M potassium phosphate buffer. When pure CO_2 gas was used as the CO_2 source, the reaction solution was purged with CO_2 gas for at least 30 mins to ensure that the solution was saturated with CO_2. The solutions of $[Ru(bpy)_3]^{2+}$, ascorbate, and bicarbonate were mixed with protein solution and buffer to a total volume of 5 mL in a GC vial containing a stir bar. The sealed

vial was transferred out of the glovebox and set up for photocatalysis. The GC vial containing the reaction mixture was mounted inside of a water bath on a stir plate. A set of 4 blue (470 nm) Luxeon Rebel LEDs were used as the light source and aligned with the sample. The distance between the LED light and the sample was about 5 cm when the GC vial was placed in the center of the water bath. The temperature of the water bath was monitored by a thermometer and found to be no more than 28 °C during the time of irradiation. All activity assays were performed in triplicate.

GC-MS/TCD/FID detection

The identification of CO as CO_2 reduction product was confirmed using Shimadzu GCMS-QP2010 Plus. To eliminate the interference of the large amount of N_2 that coelutes with CO and has a same m/z=28, we used ^{13}C-labeled sodium bicarbonate as the source of $^{13}CO_2$ substrates, with a calculated MW of 45, and the selected ion monitoring (m/z=17, 29, and 45) to look for the presence of ^{13}CO. A reaction mixture containing $[Ru(bpy)_3]^{2+}$, and CoMb was prepared in a 5 mL RB flask with a stir bar. The solution was saturated with $^{13}CO_2$ by adding sodium bicarbonate and then mounted in front of 4 blue LEDs on top of a stir plate. The solution was irradiated with stirring and the temperature was controlled using a water bath. Using a gas-tight syringe (Hamilton), 500 µL of the headspace was sampled at 30 and 60 mins and injected into the GC-MS. An air sample and a headspace sample from a control reaction where everything but protein was added were also injected into the GC-MS to serve as controls.

The headspace samples were collected from the GC vial using a gastight syringe and measured by a Shimadzu GC-2030 equipped with a flame ionization detector (FID) and a thermal conductivity detector (TCD). The detector response to CO or H_2 was calibrated using an analytical gas standard containing 1% by mols each of CO, CH_4, O_2 and CO_2 in N_2 (**Figure S4**). To ensure the consistency and reproducibility of our gas injections, we injected standard gas mix samples each day before injecting the headspace gas samples to make sure the peak areas were consistent between different days. The number of mols of CO or H_2 in the standard gas injections was calculated by first rearranging the ideal gas equation. Turnover number (TON) was calculated by dividing the mols of CO or H_2 gas detected by the mols of protein. The product selectivity towards CO generation was calculated according to equation (1):

$$\mathrm{Sel}_{\mathrm{CO_2}}=\frac{\mathrm{TON(CO)}}{\mathrm{TON(CO)+TON(H_2)}}\times 100\% \quad (1)$$

Quantum yield calculation

The overall quantum yield of the process of CO generation (Φ_{CO}) was calculated using equation (2):

$$\Phi_{\mathrm{CO}}=\frac{\text{number of CO molecules}\times 2}{\text{number of photon absorbed}}\times 100\% \quad (2)$$

The number of CO molecules was determined from the number of moles of CO in the headspace sample as detected by GC-FID. The number of photons absorbed was estimated by considering the incident light wavelength as 470 nm, the light power as 24 mW/cm^2 (measured by an optical power meter), and the illuminated area as 6 cm^2. Under the examined conditions for WT Mb, the obtained quantum yield is 0.26% at 2 h of irradiation.

Characterization of liquid-phase products

After the photo-irradiation, the solution was frozen in liquid nitrogen and then lyophilized to remove water. The solid was dissolved in D_2O and 1mM sodium 4,4-dimethyl-1-silapentane-1-sulfonate (DSS) was added as the internal standard for ^1H NMR analysis. The amount of formate was calculated by comparing the integration area of the peak at 8.5 ppm to that of the DSS peak at 0.0 ppm. All NMR spectra were acquired on a 400 MHz Bruker NMR spectrometer.

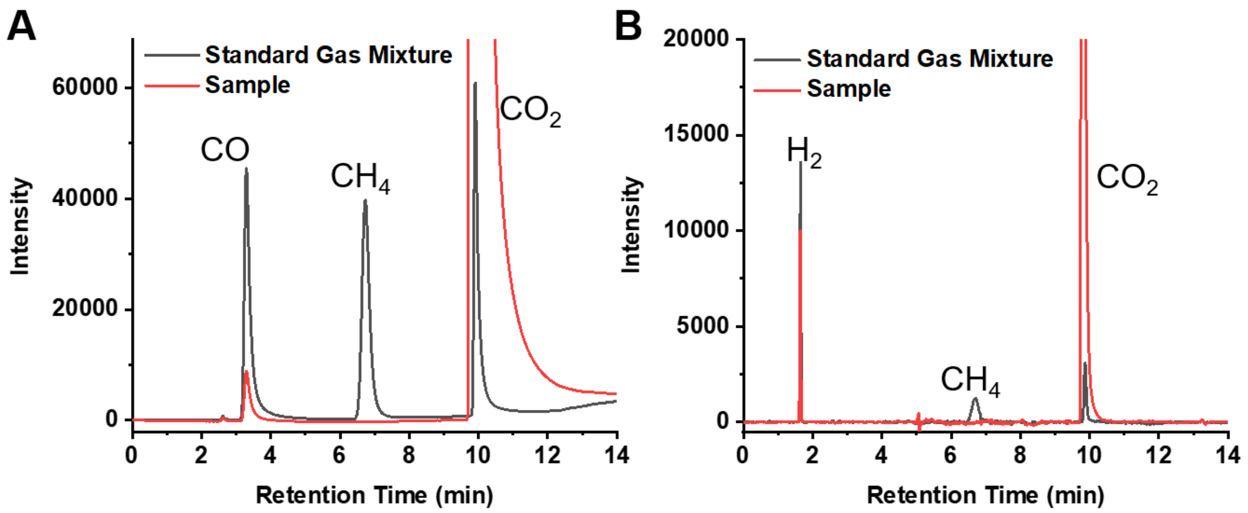

Figure S1. GC chromatograms from FID (A) and TCD (B) detectors of 100 µL standard gas mixture of CO, CH_4, CO_2, 1% each in N_2 (black) and 100 uL headspace sample from CO_2 reduction activity assay with 0.1 µM CoMb upon irradiation with blue LED lights for 30 min in 1 M potassium phosphate pH 6 buffer (red).

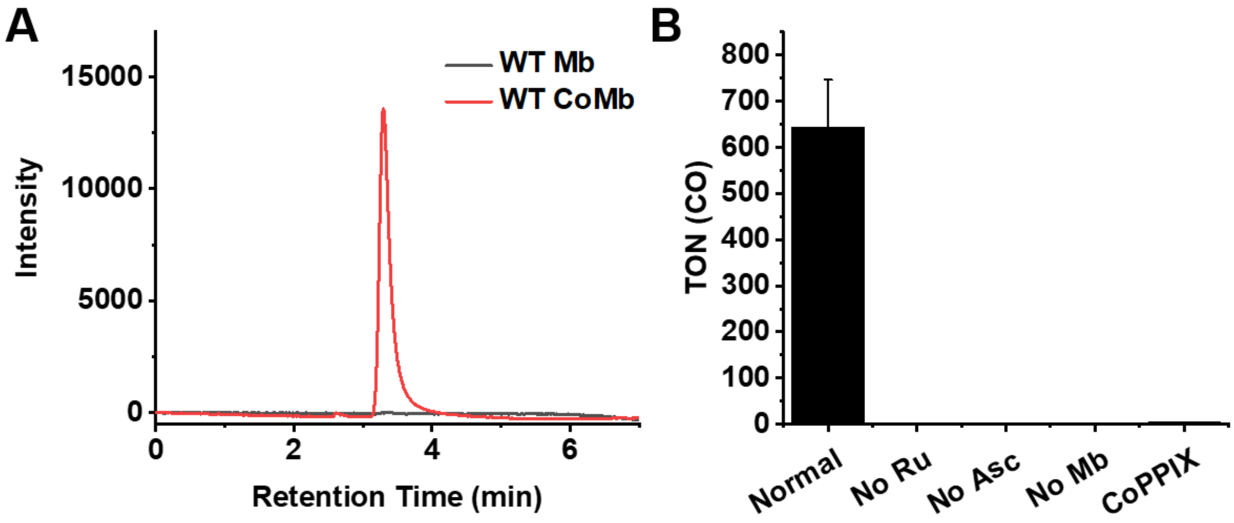

Figure S2. (A) GC chromatograms of 100 uL headspace samples from CO_2 reduction activity assays with 0.1 μM WT Mb or CoMb upon irradiation with blue LED lights for 60 min in 1 M potassium phosphate pH 6 buffer. (B) CO evolution activity obtained for CoMb upon irradiation with blue LED lights for 60 min in 1 M potassium phosphate pH 6 buffer under varying assay conditions. The "normal" assay condition indicates 100 mM ascorbic acid, 1 mM $[Ru(bpy)_3]^{2+}$, 100 mM bicarbonate, and 0.1 μM enzyme with the sample-to-LED distance being around 5 cm.

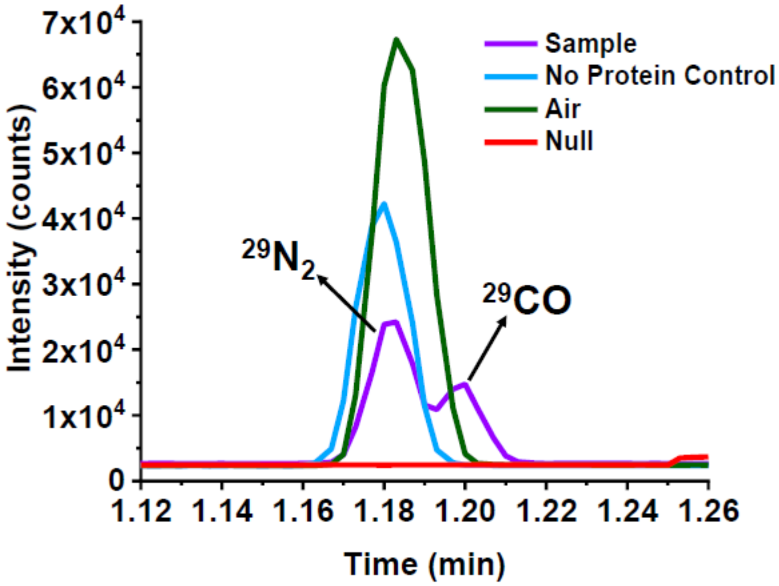

Figure S3. GC-MS chromatogram obtained after injecting a headspace sample from a 5 mL round bottom flask containing 1 mM $[Ru(bpy)_3]^{2+}$,10 mM ascorbate, $^{13}CO_2$, CoMb that was photo-reduced for 30 mins (purple trace). The species that elute with retention time ~ 1.2 mins is overlayed with controls that contain no protein (blue), a sample of air (green), and a null injection serving as the baseline (red). The major peak in the mass spec for all the chromatographic peaks shown is m/z = 29.

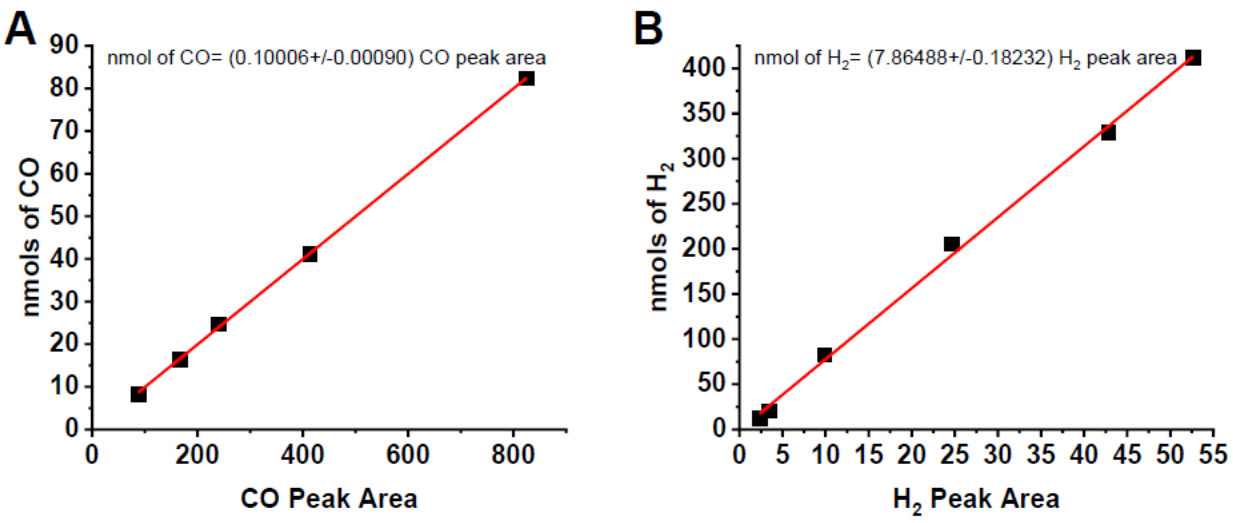

Figure S4. CO (A) and H_2(B) calibration curves constructed by injecting variable volumes of a gas standard into the GC-FID or GC-TCD and plotting the observed peak area versus calculated mols of CO or H_2.

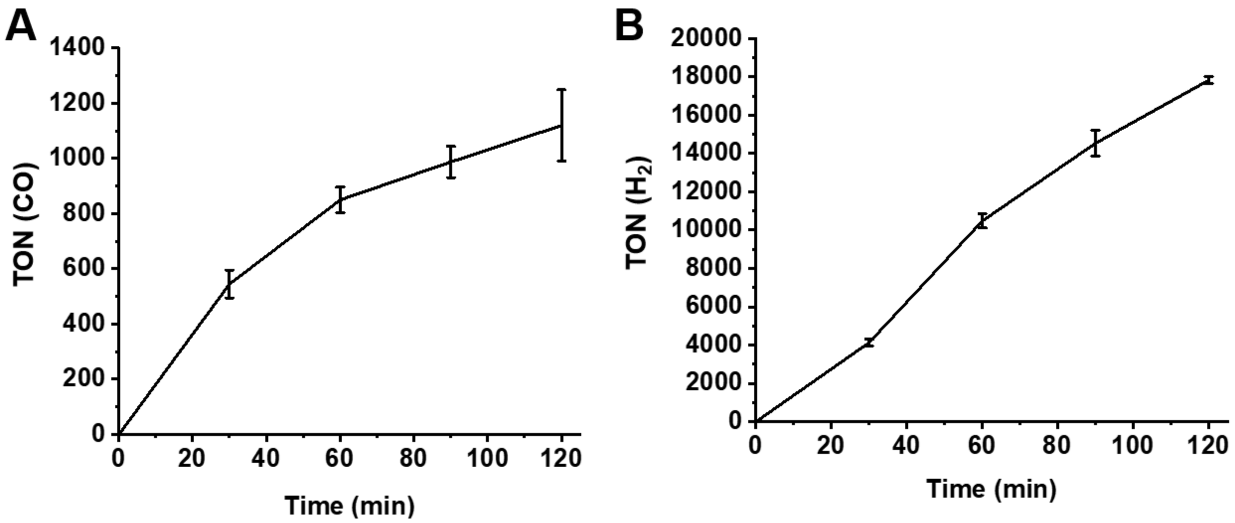

Figure S5. TON of CO (A) and H_2 (B) over time during the photoinduced reduction by WT CoMb in the presence of 100 mM sodium ascorbate, 1 mM $[Ru(bpy)_3]^{2+}$, and 1 M potassium phosphate buffer at pH 6.

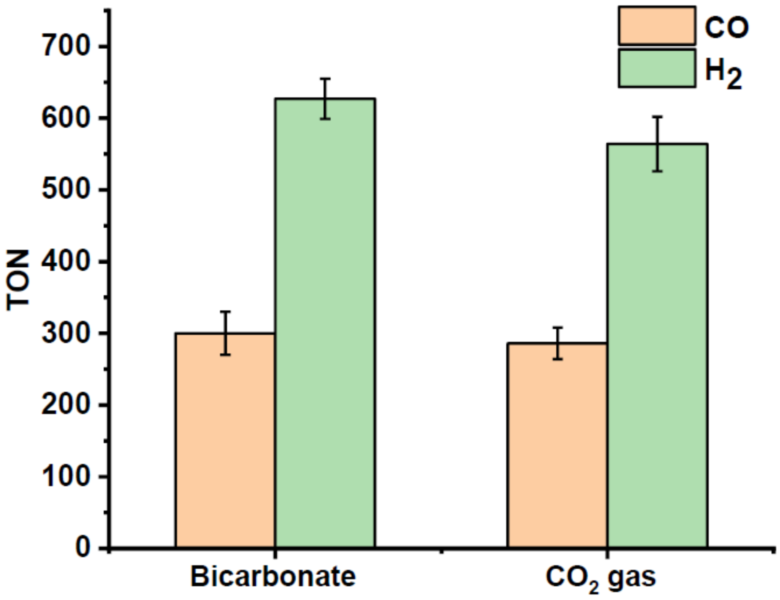

Figure S6. TON of CO (orange) and H_2 (green) production under irradiation with blue LED light for an hour in 100 mM ascorbic acid, 1 mM $[Ru(bpy)_3]^{2+}$, and 1 M potassium phosphate buffers at pH 8 using sodium bicarbonate or CO_2 gas as the source of CO_2 substrates.

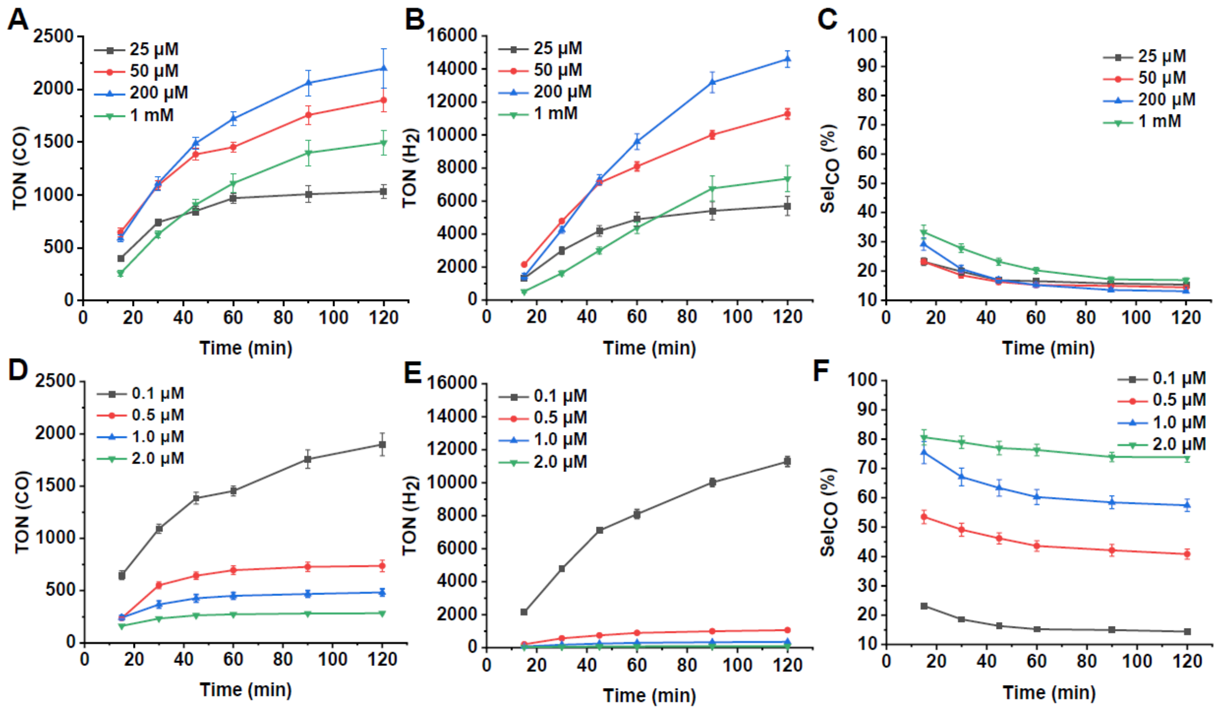

Figure S7. TON of CO and H_2, and Sel_{CO} over time during the photoinduced reduction by WT CoMb in the presence of sodium ascorbate and $[Ru(bpy)_3]^{2+}$ at pH 7. (A-C) The concentration of $[Ru(bpy)_3]^{2+}$ was varied from 25 μM to 1 mM, while the enzyme concentration was 0.1 μM. (D-F) The concentration of enzyme was varied from 0.1 μM to 2 μM, while the $[Ru(bpy)_3]^{2+}$ concentration was 50 μM.

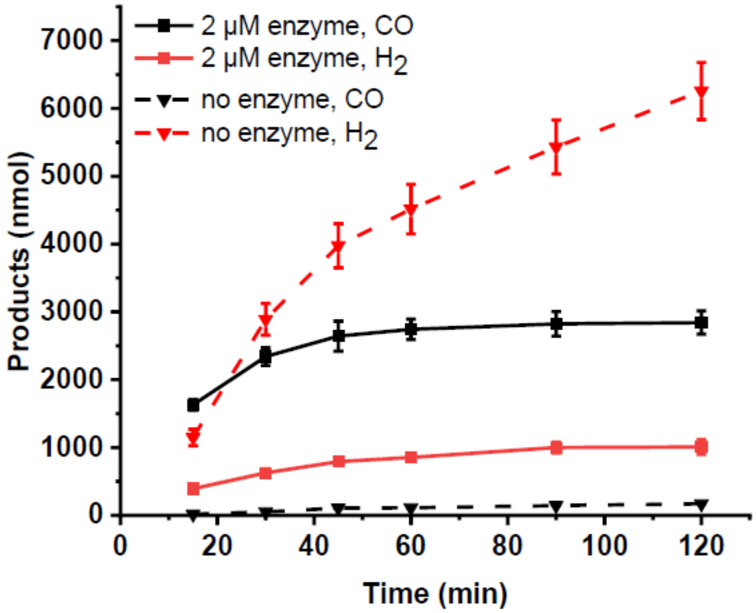

Figure S8. TON of CO (black lines) and H_2 (red lines) over time during the photoinduced reduction in the presence of 0 μM (dashed lines) or 2 μM (solid lines) WT CoMb, 100 mM sodium ascorbate, 50 μM $[Ru(bpy)_3]^{2+}$, and 1 M potassium phosphate buffer at pH 7.

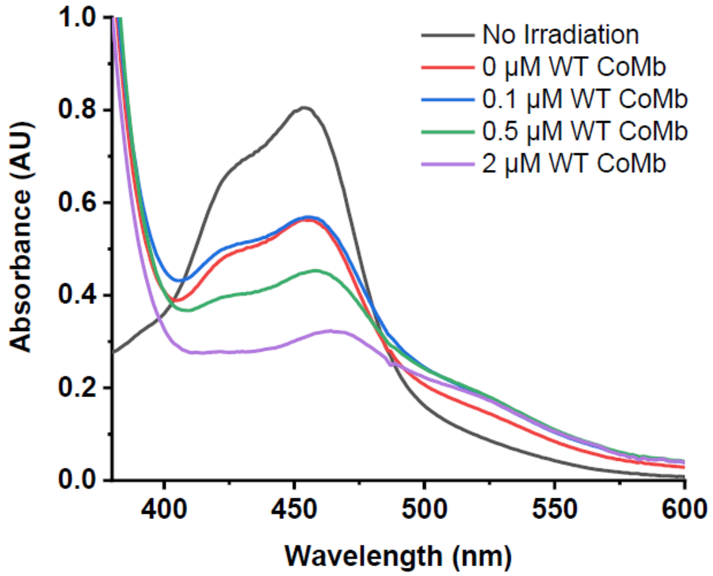

Figure S9. Electronic absorption spectra of the reaction solution with varying concentrations of the enzyme before and after 2 h photoirradiation. The initial concentration of $[Ru(bpy)_3]^{2+}$ was 50 μM.

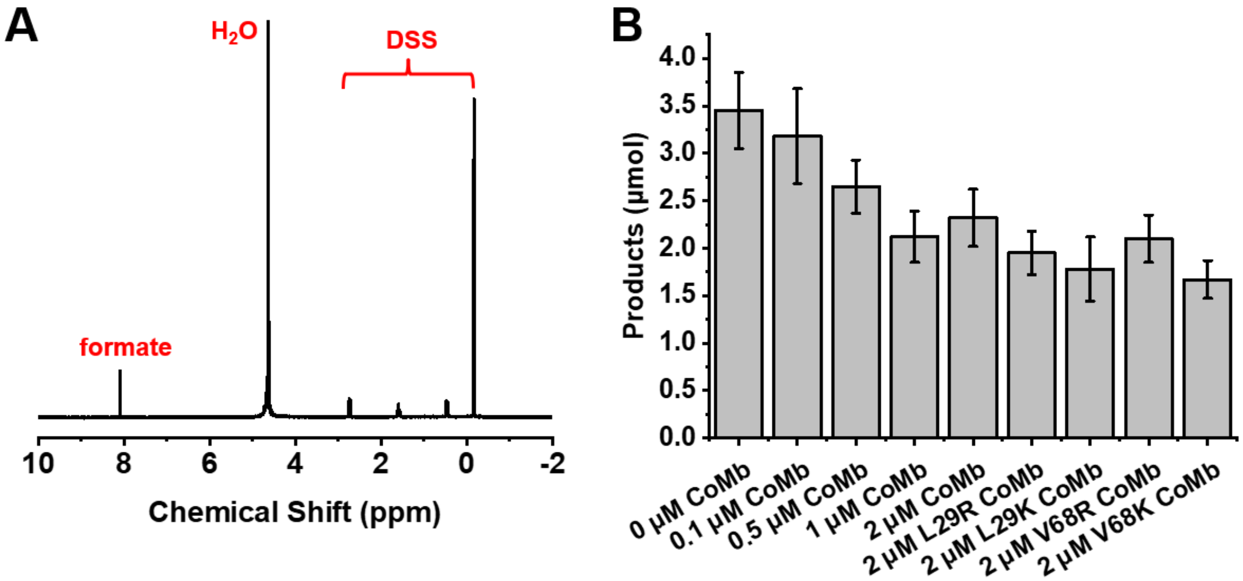

Figure S10. (A) ^{1}H NMR spectrum of formate in D_2O containing 1 mM DSS as internal standard. (B) Production of formate in the solution phase after 2 h irradiation. The reaction condition was 0-2 μM WT CoMb or mutant, 100 mM sodium ascorbate, 50 μM $[Ru(bpy)_3]^{2+}$, and 1 M potassium phosphate buffers at pH 7.

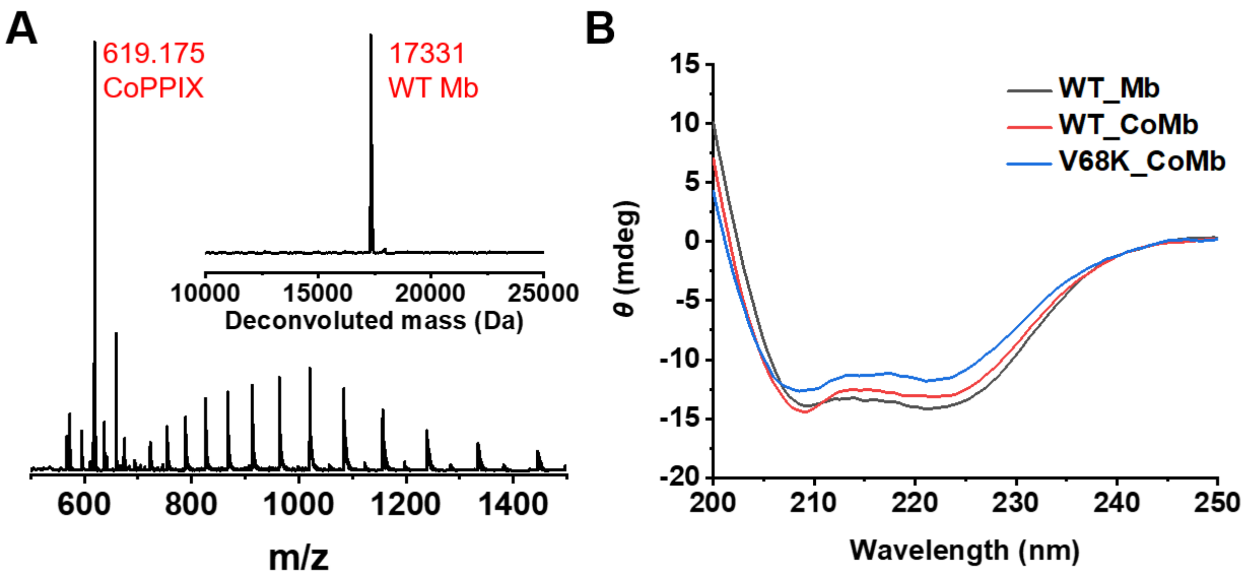

Figure S11. (A) High-resolution ESI-MS of WT CoMb (inset: deconvoluted mass spectrum, Mcalc.=17331.09 Da). (B) Far-UV CD spectra of 3 μM WT Mb (black), WT CoMb (red), and V68K CoMb (blue) in a 50 mM potassium phosphate buffer at pH 7 and 20 °C.

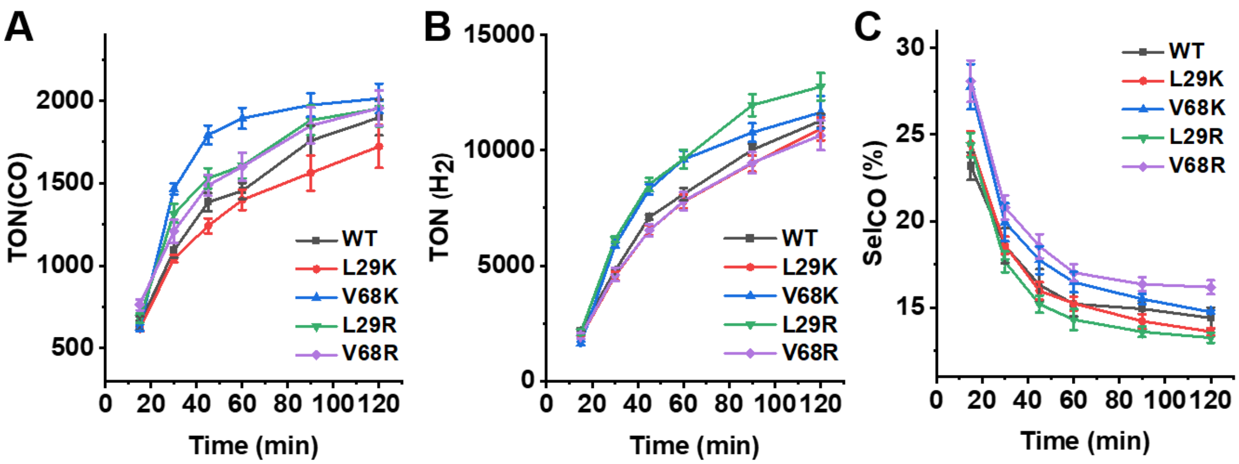

Figure S12. Mutational effects under high TON conditions. TON of CO (A), H_2 (B), and Sel_{CO} (C) over time during the photoinduced reduction by WT CoMb and variants in the presence of 0.1 μM enzyme and 50 μM $[Ru(bpy)_3]^{2+}$ at pH 7.

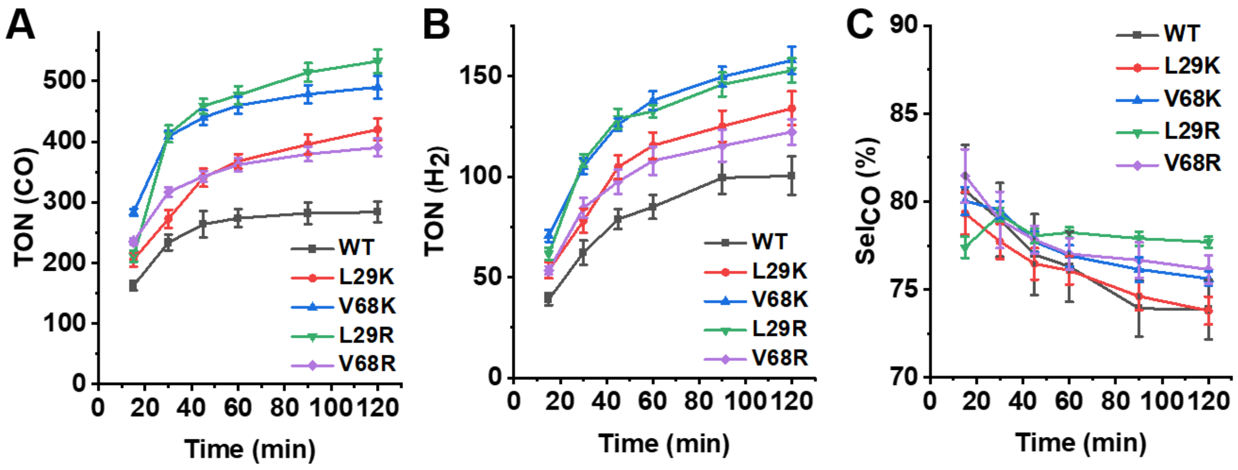

Figure S13. Mutational effects under high Sel_{CO} conditions. TON of CO (A), H_2 (B), and Sel_{CO} (C) over time during the photoinduced reduction by WT CoMb and variants in the presence of 2 μM enzyme and 50 μM $[Ru(bpy)_3]^{2+}$ at pH 7.

Table S1. TON for CO and H_2 evolutions, and product selective ratio (Sel_{CO}) for all CoMb variants after 2h photoirradiation at pH 7 under high TON conditions (0.1 μM enzyme and 50 μM $[Ru(bpy)_3]^{2+}$)

Mutant	TON(CO)	TON(H_2)	Sel_{CO} (%)
WT CoMb	1900 ± 100	11300 ± 300	14.4 ± 0.6
L29K CoMb	1700 ± 100	10900 ± 500	13.6 ± 0.2
L29R CoMb	2000 ± 100	12800 ± 600	13.3 ± 0.3
V68K CoMb	2010 ± 90	11600 ± 700	14.8 ± 0.1
V68R CoMb	2000 ± 100	10600 ± 600	16.2 ± 0.4
V68D CoMb	1700 ± 200	9400 ± 1000	15.1 ± 0.1

Table S2. TON for CO and H_2 evolutions, and product selective ratio (Sel_{CO}) for all CoMb variants after 2h photoirradiation at pH 7 under high Sel_{CO} conditions (2.0 μM enzyme and 50 μM $[Ru(bpy)_3]^{2+}$)

Mutant	TON(CO)	TON(H_2)	Sel_{CO} (%)
WT CoMb	280 ± 20	100 ± 10	73.9 ± 1.7
L29K CoMb	420 ± 20	134 ± 9	73.8 ± 0.8
L29R CoMb	530 ± 20	153 ± 6	77.7 ± 0.3
V68K CoMb	490 ± 20	158 ± 7	75.6 ± 0.4
V68R CoMb	390 ± 20	122 ± 6	76.1 ± 0.8
V68D CoMb	250 ± 30	98 ± 4	71.8 ± 1.8

References

[1] A. Bhagi-Damodaran, M. A. Michael, Q. Zhu, J. Reed, B. A. Sandoval, E. N. Mirts, S. Chakraborty, P. Moënne-Loccoz, Y. Zhang, Y. Lu, *Nat. Chem.* **2017**, *9*, 257–263.

[2] F. W. Teale, *Biochim. Biophys. Acta* **1959**, *35*, 543.

[3] M. Ikeda-Saito, T. Iizuka, H. Yamamoto, F. J. Kayne, T. Yonetani, *J. Biol. Chem.* **1977**, *252*, 4882–4887.

[4] H. Wójtowicz, M. Bielecki, J. Wojaczyński, M. Olczak, J. W. Smalley, T. Olczak, *Met. Integr. Biometal Sci.* **2013**, *5*, 343–351.
